# Prevalence of obesity and association between body mass index and different aspects of lifestyle in medical sciences students: A cross‐sectional study

**DOI:** 10.1002/nop2.638

**Published:** 2020-10-05

**Authors:** Armin Aslani, AmirReza Faraji, Bager Allahverdizadeh, Azita Fathnezhad‐Kazemi

**Affiliations:** ^1^ Student Research Committee Islamic Azad University Tabriz Branch Tabriz Iran; ^2^ Department of Midwifery Faculty of Nursing and Midwifery Islamic Azad University Tabriz Branch Tabriz Iran

**Keywords:** eating, lifestyle, obesity, physical activity, stress

## Abstract

**Aim:**

The global obesity pandemic is a major health problem with adverse effects on physical and mental health. The aim of this study was to investigate the prevalence of obesity and the association between BMI and different aspects of lifestyle.

**Design:**

A cross‐sectional study.

**Methods:**

Data collected from 380 medical sciences students using demographic characteristics and Eating Behavior, Physical Activity and Perceived Stress Questionnaires were analysed using descriptive and inferential statistics, namely analysis of variance (ANOVA), *t* test, Pearson's test and multivariate linear regression model.

**Results:**

The prevalence of obesity and overweight was 3.2% and 25.3%, respectively. There was a positive and significant statistical association between emotional eating (*r* = .542), extrinsic eating (*r* = .488) and perceived stress (*r* = .489) with BMI, also significant and an inverse association was obtained between emotional eating (*r* = −.488) and total physical activity score (*r* = −.394) with BMI. Factors such as sex, total physical activity score and leisure time activity, external eating behaviours, emotional eating, restricted eating and perceived stress had a significant role in explaining BMI changes.

**Conclusion:**

There is a need to develop interventions to improve dietary behaviours, management stress and access to sports facilities by health‐promoting activities and the provision of online health resources.

## 1 INTRODUCTION

Obesity is widely regarded as a major global pandemic (Chooi et al., 2019; Tomiyama, 2019), It is associated with numerous comorbidities such as increased cardiovascular diseases and diabetes (Blüher, 2019; Mokdad et al., 2003). Obesity has debilitating effects on both physical and mental health (Hunot et al., 2016; Jafari‐Adli et al., 2014), Finally, it leads to lower life expectancy and quality of life (Jafari‐Adli et al., 2014). The prevalence of obesity [index (BMI) over 30 kg/m^2^] has been increasing globally among different age groups and among young people (Salarzadeh Jenatabadi et al., 2020), and more than 1.9 billion adults, 18 years and older, were overweight and of these over 650 million were obese (O'Brien et al., 2016; Sanders et al., 2015). Studies in Eastern Mediterranean countries indicate that obesity has increased at an alarming level among adults (Musaiger, 2011), and the prevalence of overweight and obesity among adults ranged from 25%–81.9% (Bakhshi et al., 2011). In Iran, a various prevalence of overweight and obesity has been reported among adults (Rahmani et al., 2015) that varied between men and women (Kiadaliri et al., 2015). The results of a meta‐analysis study in Iran indicated that the prevalence of obesity has increased and the prevalence of obesity was 21.7 per cent in adults (Rahmani et al., 2015). According to the study, half of the population will be affected by obesity and overweight by 2030 (Salarzadeh Jenatabadi et al., 2020; Yang et al., 2019), and based on the WHO targets, the prevalence of obesity should be stopped by 2025, so it recommends strict monitoring of the prevalence of obesity in all populations (Johnson et al., 2006). Obesity is a multifactorial disease (Chooi et al., 2019), and the recent increase in the prevalence of obesity rates illustrates that it is associated with lifestyle factors, biological, psychosocial and familial factors (Ortiz et al., 2019; Sepulveda et al., 2019). Reduced level of physical activity, as an important problem of urbanization, and studies in medical students showed that only 26.5% of them had regular physical activity during 1 week (Ogden et al., 2015), and there was a high prevalence of inactivity among students (Mourtakos et al., 2015; Ogden et al., 2015). In addition, obesity is a major eating disorder worldwide, and due to unhealthy eating patterns, we faced an increase in obesity and overweight (Kremmyda et al., 2008). According to the studies, students tend to eat high‐fat and high‐energy snacks and skip meals such as breakfast (Sobhi & Parsamanesh, 2013; WY & AS, 2011). Furthermore, stress may affect human eating habits (Charmandari et al., 2005; Tomiyama, 2019; Yamamoto et al., 2011) and reduces the consumption of food or leads to overeating, although the severity of stressors can affect the rate of changes (Tomiyama, 2019). Long‐term stress to be associated with an increased interest in consuming high‐energy foods, such as high‐sugar and high‐fat foods (Dahlin et al., 2005). Unfortunately, perceived stress is very high among medical students (Van Jaarsveld et al., 2009; Richardson et al., 2015); although the data are inconsistent (Bose et al., 2009), It seems that stress may be related to the prevalence of obesity (Rahimibashar & Motahari, 2013). Since transition from adolescence to adulthood is a critical period for adopting health‐related behaviours to prevent illness, and medical students play an important role in promoting community health, and despite conflicting results in studies a regional difference lifestyle in different provinces of Iran; we designed a study to investigate the prevalence of obesity and overweight and association between BMI and eating habits, physical activity and perceived stress among medical sciences students.

## 2 MATERIALS AND METHODS

### 2.1 Study design

This cross‐sectional study was carried out in Tabriz Islamic Azad University of Medical Sciences from March–October 2019. Inclusion criteria were lack of history of medical problems and disability, not being a diet during the last month and no experience of stressful events during the past 6 months. Exclusion criteria were unwillingness to participate in the study and completing the questionnaire.

### 2.2 Sample size and sampling

#### 2.2.1 Sample size determination

After controlling for sample size for research purposes to determine sample size, the highest sample size was calculated based on the first objective of the study. So, considering the 11% prevalence of obesity (*p* = 11%) among medical students in the study of Mohammadi et al. in Yazd (Mohammadi et al., 2015) and with the power of 80% and the acceptable error of 0.027 around the prevalence, with using the calculation formula of proportions and after correction for the finite population of the sample were estimated to be 340, and finally, with a 10% drop in the final sample size so concidering to be 380 students.

$$n=\left(z_{1\frac{\alpha }{2}}\right)\times p\left(1-p\right)/d^{2}$$

Sampling was done according to the inclusion criteria after obtaining permission to conduct research and coordinate with the relevant authorities. In the present study, the sampling was two‐stage stratified random sampling. A proportion was chosen from each field of study in terms of sample size and then randomly sampled using the computer with Randomizer software. In all sampling stages, priority was given to those who were selected by the field quota as the main participants in the list. The researcher called the selected potentially eligible participants via phone numbers accessed from the student records and explained the objectives and methods of the study, reassured them of confidentiality of information and scheduled an appointment to attend the campus to complete the study questionnaires. After contacting the participants if the sample did not meet the inclusion criteria, either unavailable or unwilling to participate in the study, the next randomized one was replaced. Then, the researcher obtained informed consent, emphasized the importance of honest answers on the questionnaire and asked study participants to complete the anonymous questionnaires in a private class. Also, the height of those without shoes was measured. Weight was also measured in light clothing, without shoes. To measure height and weight, we used the scales of the Seka model (Germany). BMI was calculated by dividing the weight in kilograms by the height in metres squared and divided into four groups: underweight (less than 18.5), normal (18.5 and 24.9), overweight (25 and 29.9) and obese (BMI > 30). Eligible people were taken until attaining the required number of participants.

### 2.3 Data collection and measurements

1. Demographic Questionnaire: The demographic variables of the students under study included age, sex, field of study, marital status, ethnicity, residence status, smoking, height and weight.
2. Duchess Eating Behavior Questionnaire (DEBQ): This questionnaire was developed by Van Strien et al. (1986) and consists of 33 items and three subscales. The subscales included 1. restricted eating with 10 items that measure the restricted eating behaviour; 2. emotional eating with 13 items that measure eating in response to emotional distress; and 3. extrinsic eating with 10 items that measure response to external signs of food. Items are rated on a 5‐point Likert scale (never = 1 to most times = 5). This questionnaire has a validity and reliability. The sum of the scores on each subscale is the raw score of that subscale. To get the benchmark score, the raw score must be divided by the number of questions answered on the same scale. If more than one question is left unanswered on each subscale, the score is void and cannot be calculated. Fenstein et al reported reliability of this tool with Cronbach's alpha between 0.8–0.95 (Van Strien et al., 1986). In Iran, researchers have confirmed the validity and reliability of the instrument (Ashrafi et al., 2015; Salehi et al., 2011).
3. Beck Physical Activity Questionnaire: This questionnaire consists of 16 questions and contains three factors including activities related to work, leisure time and sport activities which should be answered on a Likert scale and has been scored (never = 1, always = 5, except for questions 2 and 13 which are scored vice versa). Questionnaire scoring is such that people with more physical activity get higher scores, the lowest score for an individual being 5 and the highest score being 15. This questionnaire was first developed by Beck et al. in 1982 for use in epidemiological studies (Beck dolphin). In the study in Iran, the reliability of the questionnaire with Cronbach's alpha was 0.70, and the internal consistency of the questionnaire was confirmed (Goharpei et al., 2014; Mehrabani & Mehrabani 2016).
4. Perceived Stress Scale (PSS): PSS is provided by Cohen et al. in 1983 with 3 versions of 4, 10 and 14 that was applied for measuring perceived stress in past 1 month. Because version 14 of Perceived Stress Scale is the full version of the questionnaire, we used version 14 in this study. Each question has 5 options that half of them are direct (0, 1, 2, 3 and 4), and the other half are reverse (4, 3, 2, 1 and 0) scoring formats. All items are based on the Likert scale (0 = never, 1 = low, 2 = moderate, 3 = much and 4 = very much) scoring. Scores are ranged between 0–56 sets. It should be noted that 7 questions as positive concepts (4, 5, 6, 7, 9, 10 and 13) are reverse‐scored (4 = never, 3 = little, 2 = moderate, much = 1 and very much = 0). Reliability of PSS test was 0.85, and internal consistency of this test was calculated from 0.84–0.86 (43, 42). The homogeneity coefficients of this questionnaire in the Iranian population were also confirmed by Harris and Mousavi with the Cronbach's alpha being 0.84 (Cohen et al., 1994; Cohen & Wills, 1985).

Reliability of the questionnaire was determined by using test–retest method after conducting a pilot study on 30 students. Both of the reproducibility (ICC = Intraclass Correlation Coefficient) and internal consistency (Cronbach's alpha coefficient) were determined for perceived stress ICC (confidence interval) was 0.97 (0.94–0.98) and Cronbach's alpha coefficient of 0.81, and for Duchess Eating Behavior Questionnaire ICC (confidence interval) was 0.98 (0.97–0.99) with Cronbach's alpha coefficient of 0.95, and for Beck Physical Activity Questionnaire ICC (confidence interval) was 0.93 (0.91–0.95) with Cronbach's alpha coefficient of 0.75.

### 2.4 Ethics approval and consent to participate

Ethical consideration took place throughout the data collection and analysis processes. A written informed consent was obtained from the students before data collection. This study has been approved by the Ethics Committee of the Azad Islamic Medical Sciences University, Tabriz, Iran (code number: IR.TBZMED.REC.1398.100).

### 2.5 Data analysis

Data were analysed using SPSS version 21. Descriptive statistics including frequency, percentage, mean and standard deviation (*SD*) were used to describe sociodemographic characteristics, perceived stress, physical activity and eating behaviour. One‐way ANOVA and independent *t* test were used to determine the relationship between sociodemographic characteristics and BMI, and the Pearson test was applied to determine the relationship between perceived stress, physical activity and eating behaviour with BMI. Independent variables with *p* < .2 entered into multivariate linear regression model. It should be noted that the concurrent entry strategy was used. Prior to performing multivariate regression analysis, regression assumptions including normality, homogeneity of variances and multiple linearity of independent variables were studied.

## 3 RESULTS

### 3.1 Sociodemographic characteristics

Data analysis on sociodemographic characteristics showed that the mean (*SD*) age of participants was 22.74 (3.11) with 18–40 years of age range. Two‐thirds of the participants were female, majority were single, and 266 (70%) were native. The mean (*SD*) of weight, height and BMI was 69.23 (13.39), 170.43 (8.06) and 23.65 (3.09), respectively, and nearly two‐thirds of particpants had normal BMI. The results showed that the prevalence of obesity and overweight was 3.2% and 25.3%, respectively. 2.4% of boys and 0.8% of girls were obese, and 16.8% of boys and 8.4% of girls were overweight. The mean weight of boys was higher than girls, and there was a significant difference between sex and body mass index (*p* < .001). But there was no significant difference with other sociodemographic characteristics (Table 1).

**TABLE 1:** Sociodemographic characteristics of participants and relationship with BMI (*N* = 380)

Variables	*N* (%)	BMI	*p*‐value
		Mean (*SD*)	
Sex^a^			
Male	147 (38.7)	25.29 (3.03)	<.001
Female	233 (61.3)	22.61 (2.65)	
Field of study^b^			
Medical	137 (36.1)	23.87 (3.27)	.270
Nursing	70 (18.4)	24.00 (2.91)	
Midwifery	55 (14.5)	23.32 (3.25)	
Laboratory sciences	58 (15.3)	23.73 (3.33)	
Anaesthesia	30 (7.9)	22.53 (2.00)	
Operating room nurse	30 (7.9)	23.41 (2.67)	
Ethnicity^b^			
Turkish	348 (91.6)	23.69 (3.07)	.296
Fars	13 (3.7)	22.69 (2.32)	
Kurdish	18 (4.7)	24.34 (4.36)	
Marital status^a^			
Single	345 (90.8)	23.57 (3.03)	.09
Married	35 (9.2)	24.48 (3.62)	

^a^ *t* test.

^b^ ANOVA.

### 3.2 Eating behaviour, physical activity and perceived stress

The results showed that the mean (*SD*) domains of Duch's eating behaviour including restricted, emotional and extrinsic eating were 30.92 (9.47), 33.42 (9.45) and 29.24 (7.05), respectively; this indicates that the above average scores are obtained in these areas. Also, the mean score of total physical activity score was moderate and the lowest score is related to the subscale of sport activities. The mean of perceived stress score was above average, and 317 (83.4%) had moderate and 39 (10.3%) severe perceived stress (Table 2).

**TABLE 2:** Eating behaviour, physical activity and perceived stress of the participants and correlation with BMI

Variables	Mean (*SD*)	Max	Min	*r*	*p*‐value
Eating behaviour					
Restricted eating	30.92 (9.47)	50	−0.488	−0.488	<.001
Emotionally eating	33.42 (9.45)	62	0.543	0.543	<.001
Extrinsic eating	29.24 (7.05)	49	0.448	0.448	<.001
Physical activity					
Total	7.43 (1.59)	12	−0.394	−0.394	<.001
Activities related to work	2.77 (0.48)	4.50	0.036	0.036	.483
Sport activities	2.07 (1.20)	5	−0.017	−0.017	.745
Leisure time	2.61 (0.61)	4.50	0.070	0.070	.172
Perceived stress	26.05 (6.02)	42	3	0.489	<.001

### 3.3 Association between eating behaviour, physical activity and perceived stress with BMI

According to Pearson's correlation analysis, there was a significant inverse association between subscale of restricted eating and BMI, and this correlation was moderate. There was also a moderate correlation with the emotional and External Eating subscales, and both subscales had a direct and significant association with BMI, that is, as the scores on these subscales increased, the BMI also increased.

Regarding physical activity, it can be said that there is a significant and inverse association between the total score of this scale and BMI. However, the relationship was weak. In other words, as the amount of physical activity increases, the body mass index decreases (*r* = −.394). However, no significant association was found between physical activity subscales and body mass index. The results also showed a significant and direct association between perceived stress and BMI, and this association was moderate (Table 2).

Results showed a strong correlation between sex, marital status, eating behaviour, total physical activity, leisure time activity and perceived stress with BMI, and 55.3% of changes in students' BMI depend on these variables (*r* = .753, adjusted *r* ^2^ = .575, *F* = 60.584, *p* < .001).

Linear regression model showed that factors such as sex, total score of physical activity and leisure time activity, eating behaviours and perceived stress had a significant role in explaining BMI (*p* = .05). And in this regard, sex, total score of physical activity and perceived stress have more influence on BMI. Based on the analysis, it can be said that in females, 70.7% of BMI is reduced, and by one‐unit increase in total physical activity and restricted eating, 73% and 81.4% of BMI are reduced, respectively. Also, by increasing one unit in emotional, extrinsic and perceived stress, 83%, 93% and 86% of BMI increased, respectively (Table 3).

**TABLE 3:** Multiple linear regression statistics for the relationship model of BMI with predictive variables

Variable	Unadjusted coefficient B	Adjusted coefficient B	CI (95%)		*p*‐value
			Lower	Upper	
Sex	−1.861	−0.293	0.170	0.094	<.001
Marital status	0.075	0.007	−0.651	0.801	.332
Perceived stress	0.133	0.295	0.094	0.170	<.001
Physical activity					
Total	−0.516	−0.266	−0.668	−0.365	<.001
Leisure time	0.381	0.075	0.020	0.741	.013
Eating behaviour					
Restricted eating	−0.061	−0.186	−0.086	−0.036	<.001
Emotional eating	0.54	0.166	0.021	0.088	.017
Extrinsic eating	0.032	0.072	−0.010	0.074	.033

## 4 DISCUSSION

In recent years, the increase in the prevalence of obesity, overweight and their physical and mental health problems has attracted much attention. The aim of this study was to determine the prevalence of obesity, overweight and their possible causes in medical students. The majority of participants were in the normal range of body mass index, with moderate score in physical activity and higher than average scores in various domains of perceived eating and stress behaviours.

### 4.1 Prevalence of obesity, overweight and association between BMI and sociodemographic characteristics

The results showed that 28.41% of the participants had BMI above 25, of which 3.2% had BMI above 30. There were more obese males than females, and there was a statistical significant difference between body mass index in both sexes. However, no statistical significant differences were found among other variables such as field of study, ethnicity and marital status with BMI. Studies in different parts of Iran have reported overall prevalence of overweight (16.34%) and obesity (3.04%) (Alavai et al., 2018). Also, in another study has been reported that the prevalence of obesity and overweight was 3.5% and 16.6%, respectively (Salem et al., 2016), that the prevalence of obesity was similar to our study; however, the prevalence of overweight was more than in our study. In the RahimiBashar and Motahari study on the other city of Iran, the prevalence of obesity and overweight among the 370 nursing student females was 20.8% and 3.4% (Rahimibashar & Motahari, 2013), respectively, that the prevalence of obesity was high among their participants. Also, studies on medical students in Sudan (Yousif et al., 2019) and Saudi Arabia (Saeed et al., 2017) have reported that a prevalence of obesity and overweight was 6.5% and 14.5%, respectively, indicating a high prevalence of obesity in these countries. However, studies in India show a lower incidence of obesity and overweight there (Anupama et al., 2017). The difference in prevalence can be related to cultural, economic and social factors that can influence lifestyle and diet and physical activity. Something that is clear is the high prevalence of obesity and overweight in males compared to the females in most studies, which has been reported in other studies (Anupama et al., 2017; Yousif et al., 2019), such as the present study (Kelishadi et al., 2008; Mohammadi et al., 2015). There was a statistical significant difference in BMI between males and females due to different factors such as boys' muscle mass and eating behaviours in the two groups. It seems that the higher prevalence of obesity in males than females can be attributed to cultural and social factors; for example, external eating is high among males. Also, some studies have reported positive associations between being married and weight gain (Coll et al., 2015; Price et al., 2018). However, others have reported different patterns among married women (Evers, 1987). In the current study, there was no statistical significant association between marital status and BMI. It seems to be related to the low sample size of married students.

### 4.2 Physical activity and its association with BMI

It is undeniable that proper exercise and physical activity have preventive effects on illness and are beneficial to health (Al‐Tannir et al., 2017). The assumption is that medical students may have better physical activity due to their high awareness of the participant. In the present study, the mean total score of the physical activity (7.7) was moderate and the lowest is the average score. It was related to the area of sports activities, which indicates lower participation in sports activities and lack of physical activity in leisure time among medical students. It can be justified, according to behaviour change theory, knowledge and awareness are not the only variables influencing health behaviour (Saeed et al., 2017). The association between total physical activity score and body mass index was statistically significant which is similar with the other studies (Goharpei et al., 2014; Vilchis‐Gil et al., 2015), but there were no statistically significant differences in the other studies were condocted in Iran.This may be due to differences in the samples under study that were studied in dormitory students and women of reproductive age. It may also be due to differences in students' lifestyle and lack of access to sports facilities and poor physical activity of women under study. In the study of Yousif in Sudan (Yousif et al., 2019), there was no correlation between physical activity and body mass index. The different results may be due to the type of questionnaire and physical activity tool used in the research. So, in this study, short form of physical activity was used.

### 4.3 Eating behaviour and its association with BMI

What comes out of this study is obtaining the high‐to‐average scores by students in different subscale of eating behaviours, and the behaviours associated with eating fast food, as well as emotional eating, with high scores among obese and overweight students. Significant positive correlation was observed between these subscales and BMI. However, the restricted eating score was inversely and significantly correlated with body mass index. In other words, people with low body mass index showed more diet restrictive behaviours (Ngan et al., 2017). These findings are similar with findings from other studies, and other studies have suggested a link between total calorie intake, quality of food eaten, high carbohydrate intake and fibre intake with BMI. Yousef showed a significant association between body mass index and eating behaviour by studying medical students (Yousif et al., 2019).

### 4.4 Perceived stress and its association with BMI

Medical students reported high levels of stress due to their stressful daily lives and high schooling. Our results showed that a high percentage of our college students had a moderate and higher perception of stress, similar to studies in Saudi Arabia (Gazzaz et al., 2018), Portugal (Pasco et al., 2012) and Pakistan (Ngan et al., 2017). Also, there was a significant association between perceived stress and body mass index. This finding is similar with studies in Australia (Torres & Nowson, 2007), the United States (Sims et al., 2008), India (Ngan et al., 2017) and Saudi Arabia (Gazzaz et al., 2018). In this study, there was a significant association between perceived stress and eating disorders and, consequently, inability to manage weight and body mass index.

## 5 LIMITATIONS

As the design of the study was cross‐sectional, it constrained the inferences of causality, while the extent to which the results could be generalized was limited and this study was based on questionnaire and self‐report, so the bias of the results cannot be ignored. Also, the present study was conducted as a single‐centre study only among medical students in a non‐governmental university, so the generalization of the results to other health disciplines in other areas of Iran is unclear and more studies conducted in students with different disciplines are needed.

## 6 CONCLUSION

The prevalence of overweight and obesity is relatively high among medical students. Most participants have high levels of stress, sedentary lifestyle and inappropriate eating behaviours, and these factors are significant correlates for explaining BMI. So that with increasing stress, inappropriate eating behaviours and decreasing physical activity, body mass index also increases.Since eating behaviours, physical activity and stress management ultimately make up a major component of adult's lifestyle, therefore, the promotion of these behaviours in adulthood can also reduce the incidence of obesity and overweight; accordingly, behavioural interventions such as eating behaviours, physical activity modifications and attention to the mental health should be implemented with the objective of modifying the quality of life in adolescents rather than improving anthropometric indicators. This study emphasizes that weight management initiatives should encompass psychological factors and use strategies, such as the promotion of healthy eating messages and physical activity promotion. Therefore, helping students to adopt a healthy lifestyle should not be neglected. Consequently, recommendations are made for practice or policymaking to improve weight and prevent obesity in adolescences. First, there is a need to develop interventions to improve the dietary behaviours, management stress and access to sports facilities by health‐promoting activities and the provision of online health resources. Second, educators and authorities should organize more enjoyable and fun sport programs in university campus which can help to increase physical activity and reduce stress. Also, to increase levels of awareness, it may be useful to adopt educational programs for improving eating behaviours and physical activity and stress management among students.

## CONFLICT OF INTERESTS

The authors declare that there is no conflict of interests.

## AUTHORS' CONTRIBUTIONS

A. A and A. FnK: Study design. A. A, AR. F and B. A: Implementation and data gathering. A. FnK and A.A: Analysis plan and first draft of this manuscript. All authors have critically read the text, contributed with inputs and revisions, and read and approved the final manuscript.

## ETHICAL APPROVAL AND CONSENT TO PARTICIPATE

Written informed consent will be obtained from each participant. This study has been approved by the Ethics Committee of the Azad Islamic Medical Sciences University, Tabriz, Iran (code number: IR.TBZMED.REC.1398.100).
